# Outcomes After Switching to Faricimab in Neovascular Age‐Related Macular Degeneration: Data From the Fight Retinal Blindness! Registry

**DOI:** 10.1111/ceo.14589

**Published:** 2025-07-27

**Authors:** Adrian Hunt, Yohei Hashimoto, Stephanie Young, Jennifer Arnold, Justin Game, Claire Hooper, Andrew Field, Richard Barry, Daniel Barthelmes, Mark Gillies

**Affiliations:** ^1^ The Save Sight Institute, Sydney Medical School The University of Sydney Sydney New South Wales Australia; ^2^ Department of Ophthalmology Westmead Hospital Westmead New South Wales Australia; ^3^ Gladesville Eye Specialists Gladesville New South Wales Australia; ^4^ Marsden Eye Specialists Parramatta New South Wales Australia; ^5^ Port Macquarie Eye Centre Port Macquarie New South Wales Australia; ^6^ Visionary Eye Specialists Hurstville New South Wales Australia; ^7^ Cairns Eye Surgery Edge Hill Queensland Australia; ^8^ Blink Eye Clinic Barton Australian Capital Territory Australia; ^9^ Department of Ophthalmology University Hospital and University of Zurich Zurich Switzerland

**Keywords:** AMD, Faricimab, macula, neovascular, switching

## Abstract

**Background:**

We aimed to describe 1‐year outcomes of eyes switched to faricimab from first‐generation vascular endothelial growth factor (VEGF) inhibitors for neovascular age‐related macular degeneration (nAMD) in routine care.

**Methods:**

Multicentre, observational study of 383 eyes tracked in the Fight Retinal Blindness! registry switched to faricimab from aflibercept 2 mg, ranibizumab, or bevacizumab between 1st January—1st August 2023 in Australia.

**Results:**

One‐year completion rates were high (335/383 [88%]). The proportion of choroidal neovascular (CNV) lesions graded as inactive increased from 39% at switch to 63% at 12 months (*p* < 0.01). Mean visual acuity (95% Confidence Interval) decreased from 70.0 (68.6, 71.5) to 68.4 (66.7, 70.1) logarithm of the minimum angle of resolution letters (both approx. 6/12). Mean treatment intervals increased from 7.2 to 10.5 weeks (*p* < 0.01). Eyes with active CNV at switch maintained mean VA −0.5 (−1.7, +0.7) letters; 50% were inactivated at 12 months. Eyes with inactive CNV at switch lost −3.5 (−5.0, −1.9) letters; 15% had reactivation at 12 months. Switchback occurred in 64/383 eyes (17%), predominantly to aflibercept 2 mg, that lost −1.9 letters without interval change at 12 months. Adverse outcomes were in keeping with previous reports, with no cases of occlusive retinal vasculitis.

**Conclusions:**

We found that faricimab inactivated a significant proportion of CNV lesions that had been active using 1st generation VEGF inhibitors, with a significant extension of the treatment interval. A small reduction in VA occurred in switchers and eyes not switched through the same period.

**Abbreviations**
AMD: age‐related macular degeneration
CI: confidence interval
CNV: choroidal neovascular membrane
EMR: electronic medical record
FRB!: Fight Retinal Blindness! registry
Hb: haemorrhage
IRB: Institutional Review Board
IRF/Hb: intra‐retinal fluid and/or haemorrhage
IRF: intra‐retinal fluid
LogMAR: logarithm of the minimum angle of resolution
mmHg: millimetres of mercury
nAMD: neovascular AMD
OCT: optical coherence tomography
Q1, Q3: first and third quartiles
SD: standard deviation
SRF only: only sign of neovascular disease activity is sub‐retinal fluid
SRF: sub‐retinal fluid
STROBE: strengthening the reporting of observational studies in epidemiology
VEGF: vascular endothelial growth factor 1st generation VEGF inhibitors = off‐label bevacizumab (1.25 mg), ranibizumab (0.5 mg) or aflibercept (2 mg)

## 1 Introduction

Faricimab (6 mg Vabysmo, F. Hoffmann‐La Roche AG, Basel, Switzerland), a bispecific antibody that binds both angiopoietin‐2 and VEGF‐A, was approved in Australia in January 2023 for neovascular age‐related macular degeneration (nAMD) based on the safety and efficacy demonstrated in treatment‐naïve patients enrolled in pivotal clinical trials [1, 2]. The TENAYA and LUCERNE trials reported equivalent vision gains, greater reductions in central subfield thickness and more durable efficacy in treatment‐naïve patients with nAMD compared with 2 mg aflibercept given 8‐weekly [1, 2]. The 48‐week outcomes with faricimab were maintained through 2 years with a flexible treatment interval of ≥ 12 weeks in approximately 80% of patients; one‐fifth of patients were unable to be extended beyond 8 weeks [1, 2].

Translating trial evidence to routine care can be challenging, especially when most of the workload in retinal practices involves maintenance of outcomes in existing patients already receiving treatment with first‐generation vascular endothelial growth factor (VEGF) inhibitors (Aflibercept, 2 mg Eylea, Bayer; ranibizumab 0.5 mg Lucentis, Genentech Inc/Novartis or off‐label bevacizumab, 1.25 mg Avastin, Genentech Inc., CA, USA/Roche, Basel, Switzerland).

It is unclear which patients in routine care are being switched to faricimab, but suboptimal responders are likely seen as immediate beneficiaries for switching when new options become available. A recent European FRB! study found that 25% of eyes with nAMD continued to require treatment at intervals of 6 weeks or less after 2 years using first‐generation VEGF inhibitors, highlighting the unmet need for more durable and efficacious treatments [3]. Reducing the treatment burden in patients with nAMD with longer‐acting agents could help lift what are generally less favourable outcomes observed in routine care compared to clinical trials [4, 5, 6, 7, 8, 9, 10].

Existing real‐world evidence regarding switching from first generation VEGF inhibitors to faricimab for nAMD has generally been quite positive, but these outcomes need to be interpreted with some caution since 70%–80% of the cohorts identified in analyses reported so far do not have follow‐up data [11, 12, 13]. For example, Khanani et al. reported 6‐month improvements of +2.7 letters after switching in only 22% of eyes (81/376) with follow‐up after only three injections [11]. The FARWIDE‐nAMD and FARETINA‐AMD groups, mining data from electronic medical record systems in the UK and USA, respectively, have reported stability of visual outcomes with extended intervals between treatments after switching to faricimab for nAMD, but again the outcomes refer to only 28% and 23% of eyes with extractable data through 1 year [12, 13]. In contrast, follow‐up in the FRB! registry only drops to similar levels (28%) after 10 years in patients tracked with nAMD [8].

This study aimed to use the higher quality observational data that are available from FRB! to identify the unique characteristics of patients being switched to faricimab in routine care and test the hypothesis that eyes switched from 1st generation VEGF inhibitors to faricimab for nAMD safely extend their treatment interval with reduced disease activity and stable visual acuity through 12 months.

## 2 Methods

### 2.1 Design and Setting

This was a multicentre observational study using Australian data from the prospectively designed Fight Retinal Blindness! registry [14]. Rather than mining data from medical records, FRB! collects data on a small core outcome dataset with fields that must all be populated at each patient visit via a web‐based interface. Each patient visit can be finalised only when the data are 100% complete and within pre‐specified ranges, meaning that the data quality in FRB! is more like that of a clinical trial. Unlike a trial, the FRB! registry is observational; participation does not interfere with treatment decisions. Participation involves an agreement from clinicians to record the clinical outcomes in > 85% of their patients with the relevant condition, making the sample tracked likely representative of routine care.

Ethical approval was granted by the Royal Australian and New Zealand College of Ophthalmologists (HREC#16.09) including the requirement for ‘opt‐out’ consent. This study adhered to the STROBE (Strengthening the Reporting of Observational studies in Epidemiology) recommendations regarding reporting of observational research, and complied with the Declaration of Helsinki.

### 2.2 Data Collection

The nAMD module of the FRB! registry tracks patients at each visit. Demographic details are recorded at the first injection visit. Core outcomes captured in the FRB! nAMD module include: Best corrected visual acuity (VA) in letters read on a logarithm of the minimum angle of resolution (logMAR) chart; intraocular pressure (mmHg); CNV activity (judged by the treating physician) in one of three mutually exclusive levels (Inactive, Active with only sub‐retinal fluid only [SRF only] or Active with intra‐retinal fluid or haemorrhage [IRF/Hb]); details of treatment given (name and dose of available intravitreal agents); procedures performed; adverse events; and reasons for discontinuation of treatment.

### 2.3 Patient Selection and Definitions

Eligible ‘Switchers’ with nAMD tracked in FRB! must have received their first dose of faricimab at their ‘Switch visit’ between 1st January 2023 (Date of approval in Australia) and 1st August 2023. Inclusion also required a history of treatment recorded in FRB! involving ≥ 3 prior injections of first‐generation VEGF inhibitors (Aflibercept, 2 mg Eylea, Bayer; ranibizumab 0.5 mg Lucentis, Genentech Inc/Novartis or off‐label bevacizumab, 1.25 mg Avastin, Genentech Inc., CA, USA/Roche, Basel, Switzerland) including at least 1 injection in the 6 months prior to switching. The study period in ‘Switchers’ was from the ‘Switch visit’ to the ‘12‐month visit’ closest to 365 days (±90 days) later. A 12‐month visit defined ‘completers’ of the study, whereas included eyes without a 12‐month visit were ‘non‐completers’ of the study.

To understand the unique characteristics of ‘Switchers’ we compared them with eyes that met all of the selection criteria applied to Switchers except for switching to faricimab, concurrently being managed by the same practitioners during the same recruitment period. No matching was performed; the ‘non‐Switchers’, like the Switchers, required a history of treatment recorded in FRB! involving ≥ 3 prior injections of first‐generation VEGF inhibitors. In ‘non‐Switchers’ we needed a ‘Comparative visit’ (akin to the ‘Switch visit’ in ‘Switchers’) that was closest to 1st April 2023 (±90 days) at which a VEGF inhibitor injection was delivered.

We stratified outcomes based on the CNV being ‘Active’ or ‘Inactive’ at the switch to faricimab since the goal of switching eyes that were active at the switch was likely to inactivate the lesion, whereas for eyes that were inactive it was likely in the hope of reducing the treatment burden. An Intention‐to‐Treat (ITT) study design was chosen to focus on the impact of the decision to switch to faricimab (≥ 1 injection), though we separately described outcomes based on whether treatment was ‘Switched back’ to 1st generation VEGF inhibitors (> 1 consecutive injection) within the 12‐month study period after switching to faricimab.

### 2.4 Outcomes

The primary outcome was activity of the choroidal neovascular membrane. Secondary outcomes included change in VA; the treatment interval between injections (immediately prior to the visit of interest); 12‐month completion; switchback; initial monthly faricimab loading with ≥ 4 injections in ≤ 105 days of switching (i.e., a maximum mean interval of 5 weeks) and ocular adverse events through 12 months.

### 2.5 Statistical Analysis

Statistical analysis was performed using R version 4.4.1 (http://www.R‐project.org/). Data were summarised with percentages, means with standard deviation (SD), means with 95% confidence intervals (95% CI), or medians with first and third quartiles (Q1, Q3) where appropriate. We identified statistically significant differences between ‘Switchers’ and ‘non‐Switchers’ with unpaired *t*‐tests, *χ* ^2^ tests and Fisher's exact tests. Outcomes at 12 months were analysed using paired tests to assess ‘before and after’ change within groups.

The last observation was carried forward for non‐completers, though the outcomes at the time of non‐completion were also described separately. 12‐month injections and visits were calculated for completers only. The injection delivered at the ‘Switch visit’ or ‘Comparative visit’ was included in the 12‐month injection counts. *χ* ^2^ tests were applied to assess the significance of change in the three CNV activity levels using a 3 × 3 contingency table.

## 3 Results

### 3.1 Characteristics and Disposition of Switchers

Around two‐thirds (27/42 [64%]) of Australian FRB! practitioners switched one or more of their patients with nAMD to faricimab between 1st January (first approval) and 1st August 2023. Of the 1305 eyes managed by those 27 practitioners during the recruitment period, the eyes selected for switching (383/1305 [29%]) were significantly younger (mean, 81.6 vs. 84.6 years; *p* < 0.01), had better VA (mean, 70.0 vs. 66.2 letters; *p* < 0.01), had more CNV activity (61% vs. 24%; *p* < 0.01), had shorter recent treatment intervals (mean, 7.2 vs. 10.9 weeks; *p* < 0.01), had received treatment for a shorter time (median, 1107 vs. 1427 days; *p* < 0.01) and had more frequently received aflibercept either initially (65% vs. 44%; *p* < 0.01) or most recently (89% vs. 65%; *p* < 0.01) than eyes that were not switched (922/1305 [71%]) compared in Table 1.

**TABLE 1:** Characteristics of Switchers compared with non‐Switchers managed by the same practitioners during the study recruitment period.

	Switchers	Non‐Switchers	*p*
Switch visit/comparative visit			
Eyes (*n*)	383	922	
Patients (*n*)	309	755	
Practitioners (*n*)	27	27	
Gender (% female)	63%	68%	0.11
Age, years, mean (SD)	81.6 (7.6)	84.6 (7.8)	**< 0.01**
VA, letters, mean (95% CI)	70.0 (68.6, 71.5)	66.2 (65.2, 67.3)	**< 0.01**
≥ 70 letters (%)	64%	55%	**< 0.01**
≤ 35 letters (%)	3%	7%	**0.02**
CNV activity			
Inactive (%)	39%	75%	**< 0.01**
SRF only (%)	32%	12%	**< 0.01**
IRF/Hb (%)	29%	12%	**< 0.01**
Unavailable (%)	0%	1%	—
Treatment interval, weeks, mean (95% CI)	7.2 (6.9, 7.6)	10.9 (10.7, 11.2)	**< 0.01**
Most recent drug			
Aflibercept (%)	89%	65%	**< 0.01**
Ranibizumab (%)	11%	35%	**< 0.00**
Bevacizumab (%)	0.3%	0%	—
Prior history available in FRB!			
Treatment duration, days, median (Q1, Q3)	1107 (424, 2073)	1427 (609, 2583)	**< 0.01**
VA change from baseline, letters, mean (SD)	+2.4 (15.8)	+1.7 (16.1)	0.47
Prior injections, median (Q1, Q3)	21 (9, 42)	22 (9, 38)	0.72
Prior visits, median (Q1, Q3)	23 (10, 47)	25 (10, 47)	0.97
Initial drug			
Aflibercept (%)	65%	44%	**< 0.01**
Ranibizumab (%)	33%	46%	**< 0.01**
Bevacizumab (%)	2%	10%	**< 0.01**

*Note*: Switch visit = First faricimab injection in ‘Switchers’ (between 1st January and August 2023), Comparative visit = the visit at which a VEGF inhibitor injection was delivered closest to 1st April 2023 (±90 days) in ‘non‐Switchers’, *n* = number, VA = Visual acuity in log MAR letters (best of uncorrected, corrected or pinhole), SD = Standard deviation, 95% CI = 95% Confidence Interval, Q1 = Quartile one, Q3 = Quartile three, CNV = Choroidal Neovascular Membrane, 3 levels of physician graded CNV activity: inactive, sub‐retinal fluid only (SRF only) or intra‐retinal fluid/haemorrhage (IRF/Hb). Prior history available in FRB! = treatment and outcomes recorded in the Fight Retinal Blindness! registry from first entry to the beginning of the present study period. *p*‐values < 0.05 are in bold.

Most ‘Switchers’ had CNV activity at switch (234/383 [61%]), although 39% of eyes were inactive when switched. All practitioners switched some eyes that were active but a little over half (15/27 [55%]) also switched some inactive eyes (149/383 [39%]) to faricimab. ‘Inactive’ eyes at switch had significantly longer mean treatment intervals (8.3 vs. 6.6 weeks; *p* < 0.01), were older (82.8 vs. 80.8 years; *p* < 0.01) and had been treated for longer (1204 vs. 1042 days; *p* < 0.01) than eyes that were ‘Active’ when switched to faricimab (Table 2).

**TABLE 2:** Characteristics of active versus inactive Switchers at switch.

	Active Switchers	Inactive Switchers	*p*
Switch visit			
Eyes (*n*)	234	149	
Patients (*n*)	209	123	
Practitioners (*n*)	27	15	
Gender (% female)	60%	69%	0.17
Age, years, mean (SD)	80.8 (7.2)	82.8 (8)	**< 0.01**
VA, letters, mean (95% CI)	69.2 (67.2, 71.2)	71.3 (69.2, 73.4)	0.36
≥ 70 letters (%)	63%	67%	0.32
≤ 35 letters (%)	3%	3%	0.33
CNV activity			
Inactive (%)	0%	100%	—
SRF only (%)	52%	0%	—
IRF/Hb (%)	48%	0%	—
Unavailable, %	0%	0%	—
Treatment interval, weeks, mean (95% CI)	6.6 (6.2, 6.9)	8.3 (7.7, 8.9)	**< 0.01**
Most recent drug			
Aflibercept (%)	88%	91%	**< 0.01**
Ranibizumab (%)	12%	9%	**< 0.01**
Bevacizumab (%)	0.4%	0%	—
Prior history available in FRB!			
Treatment duration, days, median (Q1, Q3)	1042 (374, 1969)	1204 (490, 2286)	**< 0.01**
VA change from baseline, letters, mean (SD)	+2.4 (16.8)	+2.3 (14.1)	0.39
Prior injections, median (Q1, Q3)	19 (8, 40)	24 (11, 43)	0.84
Prior visits, median (Q1, Q3)	20 (9, 43)	27 (13, 53)	0.52
Initial drug			
Aflibercept (%)	63%	67%	**< 0.01**
Ranibizumab (%)	34%	32%	**0.04**
Bevacizumab, %	3%	1%	**< 0.01**

*Note*: Switch visit = First faricimab injection in ‘Switchers’ (between 1st January and August 2023), *n* = number, VA = Visual acuity in log MAR letters (best of uncorrected, corrected or pinhole), SD = Standard deviation, 95% CI = 95% Confidence Interval, Q1 = Quartile one, Q3 = Quartile three, CNV = Choroidal Neovascular Membrane, 3 levels of physician graded CNV activity: inactive, sub‐retinal fluid only (SRF only) or intra‐retinal fluid/haemorrhage (IRF/Hb). Prior history available in FRB! = treatment and outcomes recorded in the Fight Retinal Blindness! registry from first entry to the beginning of the present study period. *p*‐values < 0.05 are in bold.

### 3.2 Outcomes in Switchers

**FIGURE 1:**
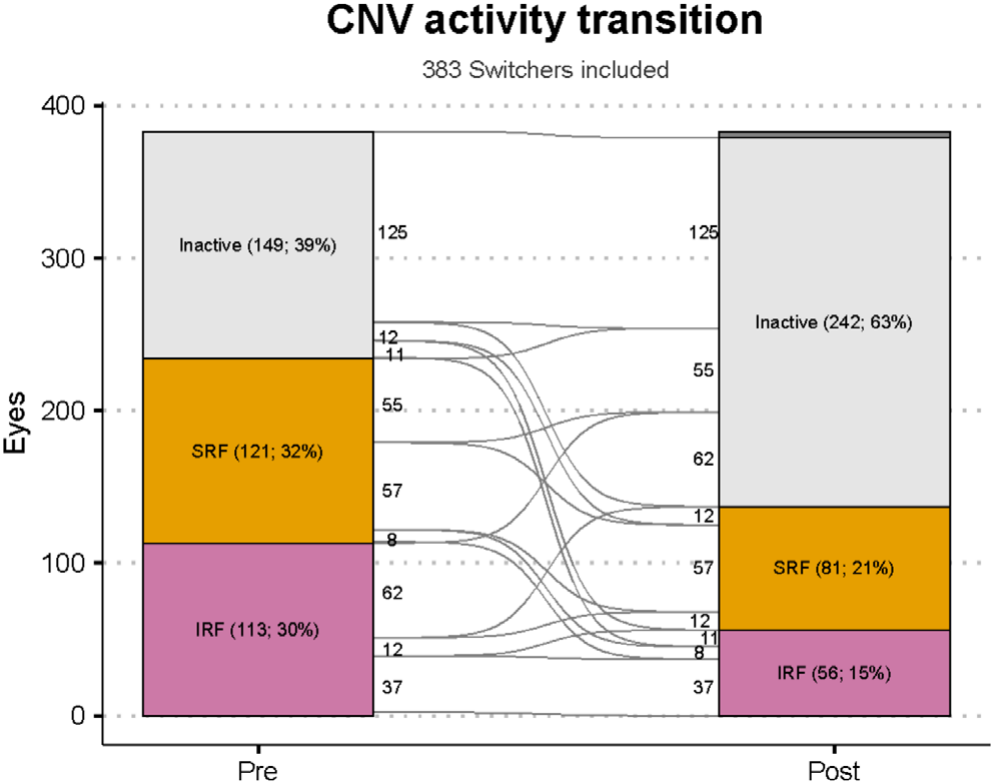
Alluvial plot demonstrating how the proportion of eyes with each category of CNV activity changed ‘Pre’ switch to 12 months ‘Post’ switch. Final CNV activity was unavailable in 3% of ‘Switchers’. In the FRB! registry, CNV activity is graded by physicians into one of three mutually exclusive categories: No activity (Inactive), subretinal fluid only (SRF), or intraretinal fluid or haemorrhage (abbreviated as IRF in this figure).

The primary outcome of CNV activity (judged by the treating physician in one of three mutually exclusive levels [Inactive, SRF only or IRF/Hb]) improved significantly 12 months after switching to faricimab (Table 3, Figure 1). The proportions of ‘Switchers’ that had CNV graded as Inactive, SRF only or IRF/Hb at ‘Switch’ were 39%, 32%, 30%, improving to 63%, 21%, 15% at 12 months (*p* < 0.01). The mean treatment interval in ‘Switchers’ increased significantly from 7.2 to 10.5 weeks (mean change [95% CI], +3.6 weeks [+2.9, +4.2]; *p* < 0.01). There was a significant change in VA 12 months after switching (mean [95% CI], −1.6 letters [−2.6 to −0.7]; *p* < 0.01).

**TABLE 3:** Twelve‐month outcomes in All Switchers and grouped by the presence of CNV activity at Switch.

	All Switchers			Active Switchers			Inactive Switchers		
	Switch	12 m	*p* (12 m Δ)	Switch	12 m	*p* (12 m Δ)	Switch	12 m	*p* (12 m Δ)
Eyes, n (12 m completion rate)	383	335 (88%)		234	204 (87%)		149	131 (88%)	
CNV activity									
Inactive (%)	39%	63%	**< 0.01** ^**b**^	0%	50%	**< 0.01** ^**b**^	100%	84%	NA
SRF only (%)	32%	21%		52%	29%		0%	8%	
IRF/Hb (%)	30%	15%		48%	19%		0%	7%	
Unavailable (%)	0%	1%		0%	2%		0%	1%	
Visual Acuity, letters									
VA, mean (95% CI)	70.0 (68.6, 71.5)	68.4 (66.7, 70.1)		69.2 (67.2, 71.2)	68.7 (66.5, 70.9)		71.3 (69.2, 73.4)	67.8 (65.1, 70.6)	
Δ VA, mean (95% CI)		−1.6 (−2.6, −0.7)	**< 0.01**		−0.5 (−1.7, 0.7)	0.36		−3.5 (−5, −1.9)	**< 0.01**
Gain ≥ 5 (%)		17%			22%			9%	
Loss ≥ 5 (%)		26%			23%			32%	
Treatment interval, weeks									
Interval, mean, (95% CI)	7.2 (6.9, 7.6)	10.5 (9.9, 11.2)		6.6 (6.2, 6.9)	9.9 (9.1, 10.7)		8.3 (7.7, 8.9)	11.6 (10.5, 12.6)	
Δ Interval, mean (95% CI)		+3.6 (2.9, 4.2)	**< 0.01**		+3.5 (2.7, 4.3)	**< 0.01**		+3.6 (2.5, 4.7)	**< 0.01**
< 8‐week (%)	60%	32%		69%	37%		46%	24%	
8 to 12‐week (%)	26%	40%		23%	39%		31%	43.9%	
≥ 12‐week (%)	14%	28%		8%	24%		24%	34%	
12 m Injections, median (Q1, Q3)^a^		7 (6, 9)			8 (6, 10)			7 (5, 8)	
12 m Visits, median (Q1, Q3)^a^		9 (7, 10)			9 (7, 11)			8 (6, 10)	
Switched‐off faricimab, *n* (%)		64 (17%)			38 (16%)			26 (17%)	

*Note*: Switch = First faricimab injection in ‘Switchers’ (between 1st January and August 2023), 12 m = 12 months (last observation carried forward in non‐completers), *n* = number, VA = Visual acuity in log MAR letters (best of uncorrected, corrected or pinhole), SD = Standard deviation, 95% CI = 95% Confidence Interval, Q1 = Quartile one, Q3 = Quartile three, CNV = Choroidal Neovascular Membrane, 3 levels of physician graded CNV activity: inactive, sub‐retinal fluid only (SRF only) or intra‐retinal fluid/haemorrhage (IRF/Hb). *p*‐values < 0.05 are in bold.

^a^ Counts were calculated in completers only, including the first faricimab injection.

^b^ *χ* ^2^ test applied to a 3 × 3 contingency table of three CNV activity levels.

The 12‐month outcomes differed based on the CNV activity of ‘Switchers’ when they switched to faricimab (Table 3). Switchers with ‘Active’ CNV at switch (The 234/383 [61%]) maintained mean VA (mean [95% CI], −0.5 letters [−1.7 to +0.7], *p* = 0.36), half became inactive (50%; *p* < 0.01, Figure 1) and their mean treatment interval extended significantly (mean [95% CI], +3.5 weeks [+2.7, +4.3]; *p* < 0.01) 12 months after switching. Switchers with ‘Inactive’ CNV at switch (39%) lost significant VA (mean [95% CI], −3.5 letters [−5.0, −1.9]; *p* < 0.01), had significant treatment interval extension (mean [95% CI], +3.6 weeks [+2.5, +4.7]; *p* < 0.01) and 15% had CNV reactivation 12 months after switching.

### 3.3 ‘Switchback’ to 1st Generation VEGF Inhibitors

Approximately 17% (64/383) of eyes included in this analysis were switched back to first generation VEGF inhibitors within the 12‐month study period. Eyes were predominantly switched back to aflibercept 2 mg (94%) at a median (Q1, Q3) of 196 days (129, 275) following faricimab treatment initiation (Table 4). During the period of receiving faricimab, there was a modest but significant increase in CNV inactivity that continued through 12 months despite switching back (Inactive at Switch/Switchback/12 months: 41%/55%/54% [12 m change: *p* < 0.01]). The VA decreased in switchback eyes while they received faricimab by a mean (95% CI) of −1.7 (−3.6, 0.1) letters; the 12‐month change after receiving faricimab and switching back was −1.9 (−3.7, −0.2) letters (*p* < 0.01 [12 m change]). The mean (95% CI) treatment interval increased modestly by +0.8 (0, +1.6) weeks while they were being treated with faricimab but dropped back to a level at 12 months that was similar to the interval when they were initially switched (12 m change +0.5 [−0.1, +1.2] weeks; *p* = 0.36).

**TABLE 4:** Outcomes in eyes that had treatment ‘Switchback’ from faricimab to 1st generation VEGF inhibitors.

	Switch visit	Switchback visit	12 m visit	*p* (12 m Δ)
Eyes, *n* (12 m completion rate)	64	64	58 (91%)	
CNV activity				
Inactive (%)	41%	55%	54%	**< 0.01** ^b^
SRF only (%)	34%	19%	18%	
IRF/Hb (%)	25%	27%	27%	
Visual Acuity, letters				
VA, mean (95% CI)	73.2 (70.6, 75.7)	71.4 (68.4, 74.5)	71.2 (68, 74.5)	
Δ VA from switch, mean (95% CI)		−1.7 (−3.6, 0.1)	−1.9 (−3.7, −0.2)	**< 0.01** ^**c**^
Gain ≥ 5 (%)		14%	13%	
Loss ≥ 5 (%)		33%	34%	
Treatment interval, weeks				
Interval, mean, (95% CI)	7.6 (6.8, 8.4)	7.9 (7, 8.8)	7.1 (6.5, 7.7)	
Δ Interval from switch, mean (95% CI)		+0.8 (0, 1.6)	+0.5 (−0.1, 1.2)	0.36^**c**^
< 8‐week (%)	53%	54%	54%	
8–12‐week (%)	28%	26%	40%	
≥ 12‐week (%)	19%	20%	6%	
Injections since switch, median (Q1, Q3)^a^		4 (2, 5)	7 (5, 9)	
Visits since switch, median (Q1, Q3)^a^		6 (4, 7)	10 (7, 13)	
Days since switch, median (Q1, Q3)		196 (129, 275)		
Switchback drug				
Aflibercept, *n* (%)		60 (94%)		
Ranibizumab, *n* (%)		4 (6%)		

*Note*: Switch = First faricimab injection in ‘Switchers’ (between 1st January and August 2023), Switchback = visit at which the treatment returned to first generation VEGF inhibitors, 12 m = 12 months after switching to faricimab (last observation carried forward in non‐completers), *n* = number, VA = Visual acuity in log MAR letters (best of uncorrected, corrected or pinhole), SD = Standard deviation, 95% CI = 95% Confidence Interval, Q1 = Quartile one, Q3 = Quartile three, CNV = Choroidal Neovascular Membrane, 3 levels of physician graded CNV activity: inactive, sub‐retinal fluid only (SRF only) or intra‐retinal fluid/haemorrhage (IRF/Hb). *p*‐values < 0.05 are in bold.

^a^ Counts were calculated in completers only and include the first injection of faricimab.

^b^ *χ* ^2^ test applied to a 3 × 3 contingency table of three CNV activity levels comparing the change from ‘Switch’ to 12 months.

^c^ *p*‐value refers to 12‐month change from the switch visit to the 12‐month visit.

### 3.4 Loading When Switching to Faricimab

Few eyes (61/383 [16%]) were initially loaded with ≥ 4 injections of faricimab in ≤ 105 days. These eyes had a mean treatment interval of 4.9 weeks before switching, which increased to 7.6 weeks at 12 months; only 13% were inactive at switch, increasing to 49% inactive at 12 months. The mean (95% CI) change in VA in faricimab‐loaded eyes was −1.6 (−4.3, 1) letters from switch to 12 months (mean final VA was 69 letters).

### 3.5 Twelve‐Month Completion After Switching

Eighty‐eight percent of ‘Switchers’ completed the 12‐month study. However, 48/383 eyes (12%) were lost to follow‐up at a median (Q1, Q3) of 156 days (90, 206) after the switch, having received a median of 3 faricimab injections. Outcomes in non‐completers at last review included reduction in CNV activity (37% inactive at switch, increased to 60% inactive at final review); mean (95% CI) change of −1.2 (−4.1, +1.6) letters since switch (mean final VA was 68 letters) and increased mean treatment intervals from 8 weeks to 12 weeks.

### 3.6 Outcomes in Eyes That Were Not Switched by the Same Physicians

We described the outcomes in ‘non‐Switchers’ through 12 months as they completed the cohort managed by included physicians through the same period (Table 5). With another year of treatment using first‐generation VEGF inhibitors, the ‘non‐Switchers’ had modest but significant improvement in CNV activity as they progressed from their comparative visit to their 12‐month visit (Inactive 75%/82%, SRF only 12%/9%, IRF/Hb 11%/8%; *p* < 0.01), significant mean (95% CI) change in −1.8 letters (−2.5, −1.2; *p* < 0.01) and significant extension of their mean treatment interval from 10.9 to 13.4 weeks (mean change [95% CI], +2.5 weeks [+2.1, +2.9]; *p* < 0.01). Relatively few of the ‘non‐Switchers’ were active (216/922 [23%]) at their comparative visit; however, 54% of these eyes became inactive at 12 months. Only 9% of the previously inactive ‘non‐Switchers’ had reactivated CNV lesions at 12 months. The significant mean loss of VA in ‘non‐Switchers’ occurred irrespective of being active (−1.6 [−3.0, −0.1] letters; *p* = 0.03) or inactive (−1.8 [−2.6, −1.2] letters; *p* < 0.01) at their comparative visit (Table 5).

**TABLE 5:** Twelve‐month outcomes in all non‐Switchers and by the presence of CNV activity.

	All non‐Switchers			Active non‐Switchers			Inactive non‐Switchers		
	Comparative visit	12 m	*p* (12 m Δ)	Comparative visit	12 m	*p* (12 m Δ)	Comparative visit	12 m	*p* (12 m Δ)
Eyes, n (12 m completion rate)	922	726 (79%)		216	168 (78%)		695	553 (80%)	
CNV activity									
Inactive (%)	75%	82%	**< 0.01** ^**b**^	0%	54%	**< 0.01** ^**b**^	100%	90%	NA
SRF only (%)	12%	9%		51%	26%		0%	5%	
IRF/Hb (%)	11%	8%		49%	20%		0%	4%	
Unavailable (%)	1%	1%		0%	0%		0%	1%	
Visual acuity, letters									
VA, mean (95% CI)	66.2 (65.2, 67.3)	64.4 (63.2, 65.6)		66.5 (64.4, 68.7)	64.9 (62.6, 67.3)		66.1 (64.9, 67.4)	64.3 (62.8, 65.6)	
Δ VA, mean (95% CI)		−1.8 (−2.5, −1.2)	**< 0.01**		−1.6 (−3, −0.1)	**0.03**		−1.8 (−2.6, −1.2)	**< 0.01**
Gain ≥ 5 (%)		18%			20%			17%	
Loss ≥ 5 (%)		31%			30%			31%	
Treatment interval, weeks									
Interval, mean (95% CI)	10.9 (10.7, 11.2)	13.4 (12.9, 13.8)		9.8 (9.3, 10.3)	11.7 (11, 12.5)		11.3 (11, 11.6)	14 (13.4, 14.4)	
Δ Interval, mean (95% CI)		+2.5 (2.1, 2.9)	**< 0.01**		+2.2 (1.4, 3)	**< 0.01**		+2.6 (2.1, 3)	**< 0.01**
< 8‐week (%)	19%	14%		32%	27%		15%	11%	
8–12‐week (%)	35%	26%		36%	25%		34%	26%	
≥ 12‐week (%)	46%	60%		33%	48%		51%	63%	
12 m injections, median (Q1, Q3)^a^		5 (4, 6)			5 (4, 7)			5 (4, 6)	
12 m visits, median (Q1, Q3)^a^		6 (5, 9)			7 (5, 9)			6 (5, 8)	

*Note*: Comparative visit = VEGF injection visit in ‘non‐Switchers’ closest to 1st April 2023 (±90 day), 12 m = 12 months (last observation carried forward in non‐completers), *n* = number, VA = Visual acuity in log MAR letters (best of uncorrected, corrected or pinhole), SD = Standard deviation, 95% CI = 95% Confidence Interval, Q1 = Quartile one, Q3 = Quartile three, CNV = Choroidal Neovascular Membrane, 3 levels of physician graded CNV activity: inactive, sub‐retinal fluid only (SRF only) or intra‐retinal fluid/haemorrhage (IRF/Hb). *p*‐values < 0.05 are in bold.

^a^ Counts were calculated in completers only, including the comparative visit injection.

^b^ *χ* ^2^ test applied to a 3 × 3 contingency table of three CNV activity levels. CNV activity was unavailable at the Comparative visit in 11/922 [1%] of non‐Switchers—these eyes were included in ‘All’ but not included in either of the ‘Active’ or ‘Inactive’ columns.

### 3.7 Adverse Outcomes

The entire population of eyes managed by included practitioners was monitored for adverse outcomes through 12 months whether they were ‘Switchers’ or ‘non‐Switchers’. A total of 7013 injections (2407 faricimab, 4606 VEGF) were delivered with adverse events and outcomes described in Table 6. Intraocular inflammation (IOI), consisting of anterior uveitis or vitritis, occurred in 6/383 ‘Switchers’ (1.6%) after their 2nd to 8th injection of faricimab (risk per injection: 0.2%) with all but one eye subsequently switching back to aflibercept 2 mg. Switchers that developed IOI while receiving faricimab maintained the same mean VA from switch to 12 months (mean 12‐month change 0 letters, all but one eye had 12‐month VA > 75 letters). One ‘non‐Switcher’ developed vitritis with aflibercept 2 mg after 100 prior aflibercept injections, losing 23 letters from their comparative visit to 12 months. Other adverse outcomes included one retinal detachment, a macular haemorrhage reducing VA ≥ 15 letters and two cases of infectious endophthalmitis, but no cases of non‐infectious endophthalmitis, non‐occlusive or occlusive retinal vasculitis, chorioretinitis or retinal pigment epithelial tear.

**TABLE 6:** Ocular adverse events, treatment and outcomes.

	Switcher or non‐Switcher	Preceding treatment	Subsequent treatment	12 m VA (letters)	12 m Δ VA (letters)
Anterior uveitis	Switcher	Faricimab#7	Faricimab	84	2
Anterior uveitis	Switcher	Faricimab#4	Aflibercept	76	−7
Anterior uveitis	Switcher	Faricimab#8	Aflibercept	47	−22
Anterior uveitis	Switcher	Faricimab#3	Aflibercept	80	17
Anterior uveitis and vitritis	Switcher	Faricimab#3	Aflibercept	83	10
Anterior uveitis and vitritis	Switcher	Faricimab#2	Aflibercept	83	0
Vitritis	Non‐Switcher	Aflibercept#101	Aflibercept	35	−23
Haemorrhage reducing VA ≥ 15 letters	Switcher	Faricimab#2	Faricimab	47	−15
Infectious endophthalmitis	Switcher	Faricimab#3	Faricimab	49	−9
Infectious endophthalmitis	Non‐Switcher	Aflibercept#7	Aflibercept	2	−66
Retinal detachment	Switcher	Faricimab#4	Faricimab	79	−1

*Note*: VA = Visual acuity, # = injection number of that agent since the first visit entered in the registry, 12 m VA = VA at 12 months or last observation carried forward in non‐completers, ΔVA = Change in VA from the switch or comparative visit to 12 months (letters).

## 4 Discussion

Around two‐thirds of Australian FRB! practitioners (27/42 [64%]) switched some of their patients with nAMD from first‐generation VEGF inhibitors to faricimab in the 7 months after listing on the Pharmaceutical Benefits Scheme in January 2023. The subset of eyes selected by those practitioners for switching to faricimab (383/1305 [29%]) differed from eyes not switched during the same period by the same practitioners (922/1305 [71%]) in a number of ways. The faricimab‐switched eyes had more active CNV lesions (61% vs. 24% active [*p* < 0.01]), had shorter treatment intervals (mean, 7 vs. 11 weeks [*p* < 0.01]), were on average 3 years younger, had received treatment for one less year, and more frequently had started with or had switched to aflibercept compared with the eyes that were not switched to faricimab.

The activity of the CNV, the primary outcome, improved significantly in eyes switched to faricimab from first‐generation VEGF inhibitors for nAMD. Half of the eyes that had active CNV became inactive, while only 15% of inactive eyes developed activity 12 months after switching to faricimab. The mean interval between injections increased significantly from 7.2 weeks to 10.5 weeks. There was a small but significant mean (95% CI) loss of −1.6 (−2.6, −0.7) letters 12 months after switching, keeping in mind that a gradual decline of approximately 1–2 letters per year in mean visual acuity once treatment has been established is common in patients with neovascular AMD managed in routine care, often attributed to progressive atrophy or suboptimal treatment intensity [8].

Most patients and their doctors would welcome improvements in anatomical outcomes and longer intervals after switching treatment. However, the small change in VA following treatment switch warrants further discussion. Specifically, it is important to consider whether such a minor change in mean VA should be viewed as a clinically meaningful loss or as a maintenance of VA. A critical aspect of non‐inferiority trials in ophthalmology is setting an appropriate non‐inferiority margin, often measured in a few letters [15]. In the TENAYA and LUCERNE trials, a 4‐letter reduction margin was used to assess the non‐inferiority of faricimab compared to aflibercept [1, 2]. The eyes that were not switched by the same practitioners lost a similar amount of VA with another year of treatment in routine care.

The eyes with CNV activity when they switched to faricimab appear to have had the best outcomes in terms of CNV inactivation, maintenance of VA and reduced treatment frequency 12 months later. The active switchers were the only group that maintained mean VA through 12 months (mean [95% CI], −0.5 letters [−1.7, 0.7]). The overall loss of VA in switchers can be largely attributed to the inactive switchers, likely motivated by potential interval extension, that had significant changes in VA at 12 months (−3.5 letters [−5.0, −1.9]).

One hope is that new agents may satisfy the unmet need for greater durability and efficacy, particularly in sub‐optimal responders receiving first‐generation VEGF inhibitors for nAMD [3]. Clinicians primarily switching sub‐optimal responders in their own practice are likely to be disappointed if they expect to emulate the treatment intervals achieved in the treatment‐naïve patients enrolled in the TENAYA and LUCERNE trials [2]. In terms of reliance upon frequent treatment, our subset of switchers likely has more in common with the 20% of trial patients unable to extend beyond 8 weeks than the 80% that were able to extend to 12 or more weeks on a personalised treatment interval in those trials [2]. Nevertheless, the 3.5‐week extension of the mean treatment interval effectively increased the period between injections by 50% for the patients in our study, which is a clinically meaningful reduction in burden for these patients and providers alike.

Seventeen percent of eyes that were switched to faricimab were switched back to a 1st generation VEGF inhibitor, typically aflibercept, at a median of 6 months. These eyes had similar loss of VA at 12 months (−1.9 letters [−3.7, −0.2] at 12 months; −1.7 [−3.6, +0.1] letters while on faricimab) to eyes that continued faricimab and eyes that did not switch at all. Even though a significant proportion of switchback eyes had inactivation of their CNV lesion at 12 months, it occurred at the lowest rate of any group. Switchback eyes were the only group that did not have a significant extension of their treatment interval at 12 months. The finding highlights a treatment gap in a subset of patients who may be resistant to both first‐generation VEGF inhibitors and faricimab. This unmet need underscores the importance of developing novel therapies.

The switchback rate in our study was somewhat similar to an American single centre study that reported 9% of eyes returned to aflibercept treatment within 6 months of switching to faricimab [16]. A much higher rate was reported in a 6‐month Japanese study of highly treatment dependent eyes (*N* = 130) that had exudation despite 4‐weekly aflibercept when switched to faricimab. The Japanese label mandated bimonthly faricimab after loading, which was not tolerated by 59% of eyes that were switched back to aflibercept or to brolucizumab because of worsening exudation [17]. We observed the rarity of loading at switch in eyes that were not previously on short treatment intervals in Australia.

The description of outcomes in the eyes not switched to faricimab added important context to this study [18]. CNV activity also reduced in non‐switched eyes and their treatment intervals extended, suggesting a broader trend possibly related to overall treatment strategies rather than the specific agent, though the effects were less than in the switched eyes. It makes more notable the maintenance of VA in active faricimab switchers, the larger reduction in VA in the inactive faricimab switchers, and the lack of interval extension seen in eyes switched back to VEGF inhibitors during the 12‐month study.

Previously active eyes had similar rates of inactivation at 12 months irrespective of switching or not switching (54%, 50%, respectively). This may be surprising but needs to be interpreted with an understanding that the mean treatment intervals also changed differently over 12 months in switchers and non‐switchers (6.6–9.9 weeks, 9.8–11.7 weeks, respectively). The treatment intervals were likely titrated through a Treat & Extend (TAE) regimen widely used by Australian practitioners to achieve similar levels of disease control.

This study occurred at a time when there is heightened awareness of intra‐ocular inflammation (IOI) associated with intravitreal therapies [19, 20, 21]. When studied head to head in treatment naïve eyes, the TENAYA and LUCERNE studies did not find a significant difference in the rates of IOI between faricimab and aflibercept 2 mg [1, 2]. The switchers in our study were being exposed to a new drug for the first time, whereas non‐switchers were continuing the same treatment for another year. The numbers were small, but this may in part explain why intraocular inflammation occurred mainly in eyes (6/7) switched to faricimab and uncommonly in non‐switchers (1/7). Five of the 6 eyes that developed anterior uveitis or vitritis while receiving faricimab were switched back to first generation VEGF inhibitors. Nevertheless, the maintenance of VA in switchers that developed IOI was reassuring.

The proportion of eyes that completed the present study was high; we had registry data that was 100% complete and within prespecified ranges available in 88% of eyes through 12 months, which provides a relatively complete account of outcomes after switching to faricimab for nAMD in routine care. Existing reports have generally been very positive regarding outcomes of switching to faricimab for nAMD but have failed to provide outcomes for 70%–80% of the patients that they studied for relatively short periods [11, 12, 13] they have described outcomes only in the eyes that stayed on faricimab for the entire study period [17, 22] or have reported outcomes in only the immediate months after switching to faricimab from first generation VEGF inhibitors [17, 23, 24, 25] The reduction in VA of 1 to 2 letters on average that we found 12 months after switching (or not switching) is likely more realistic and in keeping with previously reported year‐on‐year trends [8]

Retrospective observational research has several inherent limitations. These may have been offset in part by the prospective design of the FRB! registry and the excellent follow‐up in the present study. The data within the registry allowed us to characterise the patients selected for switching but not the clinical reasoning behind that decision. We cannot be sure that consecutive patients were entered in the registry by physicians even though it is a condition of participation. Most studies endeavour to start with similar groups exposed to different treatments. The ‘Switchers’ in our study were selected non‐randomly; they differed significantly from the ‘non‐Switchers’, meaning that outcomes 12 months later cannot be attributed to switching alone. We have also been cautious comparing outcomes such as treatment intervals in our select cohort of switchers with those of trial patients that were treatment‐naïve, amongst other strict inclusion criteria [1, 2]. Nevertheless, the results of our study are likely generalisable to patients with nAMD in routine care reliant on frequent treatment with first generation VEGF inhibitors that may be considering switching to faricimab.

This study using high quality data from the Fight Retinal Blindness! registry in Australian patients found that eyes that switched treatment from first generation VEGF inhibitors to faricimab for nAMD had significantly better anatomical outcomes, lower burden of treatment through treatment interval extension but with a small mean VA loss 12 months after switching. Eyes with CNV lesions that were active at the switch appeared to benefit most as they maintained VA, whereas eyes that were inactive at the switch (29%) lost a small amount of VA after switching. A small proportion (17%) of eyes switched back to first generation VEGF inhibitors (94% to aflibercept). We observed a risk of 0.2% per injection of IOI during a period of heightened vigilance. Our results support consideration of switching treatment to faricimab in eyes with nAMD that are responding sub‐optimally to 1st generation VEGF inhibitors.

## Conflicts of Interest

M.C.G. and D.B. are inventors of the software used to collect the data for this analysis. The following authors are members of the advisory boards of Roche (A.H., M.G.); Bayer (M.G.); Allergan (M.C.G.). Honoraria were reported from Bayer and Roche (AH), travel expenses from Bayer (A.H., A.F.), D.B. received research grants from Novartis and Bayer and is a consultant to Alcon, M.G. has received research funding from Bayer, Roche and Apellis.

## Data Availability

Research data are not shared.
